# Efficacy of intravenous acetaminophen on postoperative shivering: A meta-analysis of randomized controlled trials

**DOI:** 10.1097/MD.0000000000038710

**Published:** 2024-07-12

**Authors:** Jikai Liu, Qian Cao, Jinfang Zeng, Xiao Liang

**Affiliations:** a School of Medicine, Jiangnan University, Wuxi, China; b Department of Anesthesiology, Jiangnan University Medical Center, Wuxi, China.

**Keywords:** acetaminophen, meta-analysis, shivering

## Abstract

**Purpose:**

Postoperative shivering (POS) is a common and vital complication after anesthesia, which may result in serious consequences and uncomfortable experiences. Acetaminophen has been used to treat fever and mild to moderate pain. However, there is not enough evidence to prove its advantage for POS. This meta-analysis aimed to explore the prophylactic use of acetaminophen as a valid agent for POS.

**Methods:**

Two researchers independently searched PubMed, the Cochrane Library, and Embase for controlled clinical trials. The meta-analysis of randomized controlled trials (RCTs) was performed by Review Manager.

**Results:**

Nine trials with 856 patients were included in our meta-analysis. Acetaminophen significantly reduced POS compared with placebo (pooled risk ratio [RR]: 0.43, 95% confidence interval [CI]: 0.35–0.52). What is more, not only 15 mg/kg but also 1000 mg intravenous acetaminophen could reduce the incidence of shivering compared with placebo.

**Conclusion:**

Our present meta-analysis demonstrates that the intravenous prophylactic infusion of acetaminophen may prevent POS, and the results may provide new evidence to expand the clinical value of acetaminophen in addition to its routine usage.

## 1. Introduction

Shivering is defined as an involuntary physiological response in skeletal muscles which can effectively preserve heat as a thermoregulatory response to cold, and it occurs both in the early stages of recovery from neuraxial anesthesia and general anesthesia, which leads to hypothermia during the procedure.^[1]^ It is a frequent side effect of anesthesia and specific targeted temperature modulation. Postoperative shivering (POS) is regarded as one of the commonest complications that occur in the recovery period, with an incidence varying from 5% to 65% in general anesthesia and 30% to 55% in regional anesthesia.^[2]^

The exact mechanisms underlying POS after anesthesia remain incompletely understood,^[3]^ what is certain is that it brings very uncomfortable experiences and may give rise to undesired consequences.^[4]^ Factors that cause core hypothermia could result in POS, such as the patient age, gender, duration of surgery, and room temperature.^[5]^ Not only could POS cause discomfort in conscious patients but it leads to more oxygen consumption and carbon dioxide production as well. What is worse, increasing catecholamine release and intracranial pressure is a threat in patients with myocardial ischemia and frailty.^[6]^ Shivering during anesthesia may interfere with the monitoring of blood pressure, electrocardiogram, and pulse oximetry, as well as reducing patient comfort and satisfaction.^[7]^ Therefore, it is particularly significance to reduce adverse reactions such as shivering during the recovery phase.

Pathogenesis of POS remains unknown, but varied drugs and methods have been applied for treatment and prevention of POS.^[8]^ Nonpharmacological measures included pre-warming the patient with a forced air warmer, and avoiding administration of cold epidural and intravenous fluids, while treatment drugs such as fentanyl, meperidine, ondansetron, ketamine, tramadol and clonidine are alternative drugs in POS.^[9]^

Acetaminophen acts through centrally mediated prostaglandin inhibition to decrease the hypothalamic temperature set point.^[10]^ Prophylactic use of intravenous acetaminophen has been used for POS after anesthesia. To the best of our knowledge, some clinical researchers have already studied the administration of acetaminophen to relieve POS. This current meta-analysis was designed to discuss the certainty of acetaminophen for POS. So, we conducted this meta-analysis aiming to explore the prophylactic use of acetaminophen as an efficacious agent for POS.

## 2. Materials and methods

We conducted a meta-analysis to assess the efficacy of intravenous acetaminophen on POS, as recommended by the PRISMA statement. The registration number of the study in PROSPERO is CRD 42023470294.

### 2.1. Search approach and eligibility standards

Two authors (Liu and Cao) independently conducted a systematic literature search by using Cochrane Central Register of Controlled Trials (CENTRAL), Embase, Web of Science and PubMed. The search strategy contains the following key words: ((((((((Acetaminophen) OR (APAP)) OR (p-Acetamidophenol)) OR (Paracetamol)) OR (Acetamidophenol)) OR (Panadol)) OR (Acamol)) AND (((((((shivering) OR (shiver)) OR (tremor)) OR (shaking)) OR (chill)) OR (rigors)) OR (ague))) AND (((((anesthesia) OR (anesthesia,)) OR (surgery)) OR (operation)) OR (postoperative)). The literature search was updated on October 10, 2023, limited to English papers only. After screening titles and abstracts, Randomized controlled trials (RCTs) were filtered out, and literature not RCTs or not related to POS was excluded.

### 2.2. Selection criteria

Inclusion criteria were as follows:

1. Studies designed as RCTs.
2. Adult patients (age ≥ 18 years) undergoing surgery under general anesthesia or spinal anesthesia.
3. Acetaminophen was performed in the experimental group, and the control group was placebo or received no intervention.
4. Outcomes such as incidence of POS; different grades of shivering, incidence of hypotension, incidence of postoperative nausea or vomiting of shivering, and hypothermia.

Exclusion criteria were as follows:

1. Non-RCTs.
2. Abstract only or not full text.
3. Reviews, letters, abstracts, editorials, or studies reporting insufficient data.
4. No control group.

### 2.3. Data extraction

Relevant data were extracted from qualified studies including the first author name, publication year, type of anesthesia and surgery, the age range of the patients, incidence of shivering, incidence of postoperative nausea and vomiting (PONV), incidence of hypotension, patients in need of antishivering treatment, duration of operation. All the data were counted on a uniform table. Divergence was settled promptly by discussion among group members.

### 2.4. Qualitative assessment

Quality of the studies was evaluated, independently, according to the guidelines of Cochrane Collaboration, containing 6 categories (randomization sequence generation (selection bias), blinding method (performance bias and detection bias), allocation concealment (selection bias), incomplete outcome data (attrition bias), selective reporting (reporting bias), and other bias, with the first 3 categories considered as “key domains”). The categories above could be summarized into 3 levels, high risk, unclear risk, and low risk. The risk of bias of each study was evaluated according to the levels of the 3 key domains: “High” (high risk of bias for one or more key domains), “Unclear” (unclear risk of bias for one or more key domains), and “Low” (low risk of bias for all key domains).

Quality of evidence was evaluated by GRADE (Grades of Recommendation, Assessment, Development, and Evaluation) system using the Guideline Development Tool. We performed quality of evidence and strength of GRADE recommendations for primary outcomes from included RCTs and they are shown in Supplementary Table 2 http://links.lww.com/MD/N184 and summarized in Supplementary Table 1. http://links.lww.com/MD/N183 Five factors contribute to the downgrade, consisting of risk of bias, inconsistency, indirectness, imprecision, and publication bias. There are 3 factors that contribute to the upgrade, consisting of large effect, plausible residual confounding, and dose-response gradient.

### 2.5. Statistical analysis

Side effects of intravenous acetaminophen such as shivering, PON, POV, PONV, and hypotension were evaluated by pooled risk ratio (RR), and core temperature before and after anesthesia was assessed by pooled standard mean difference, with 95% confidence intervals (CI). Z test (*P* < .05 was considered statistically significant). The fixed effect model was used when I^2^ ≤ 50%, and the random effect model was used otherwise. Sensitivity analyses reanalyzed only data from studies with low risk and uncertain risk to test the robustness of these results. Subgroup analyses were performed according to dosage.

## 3. Results

**Figure 1.**
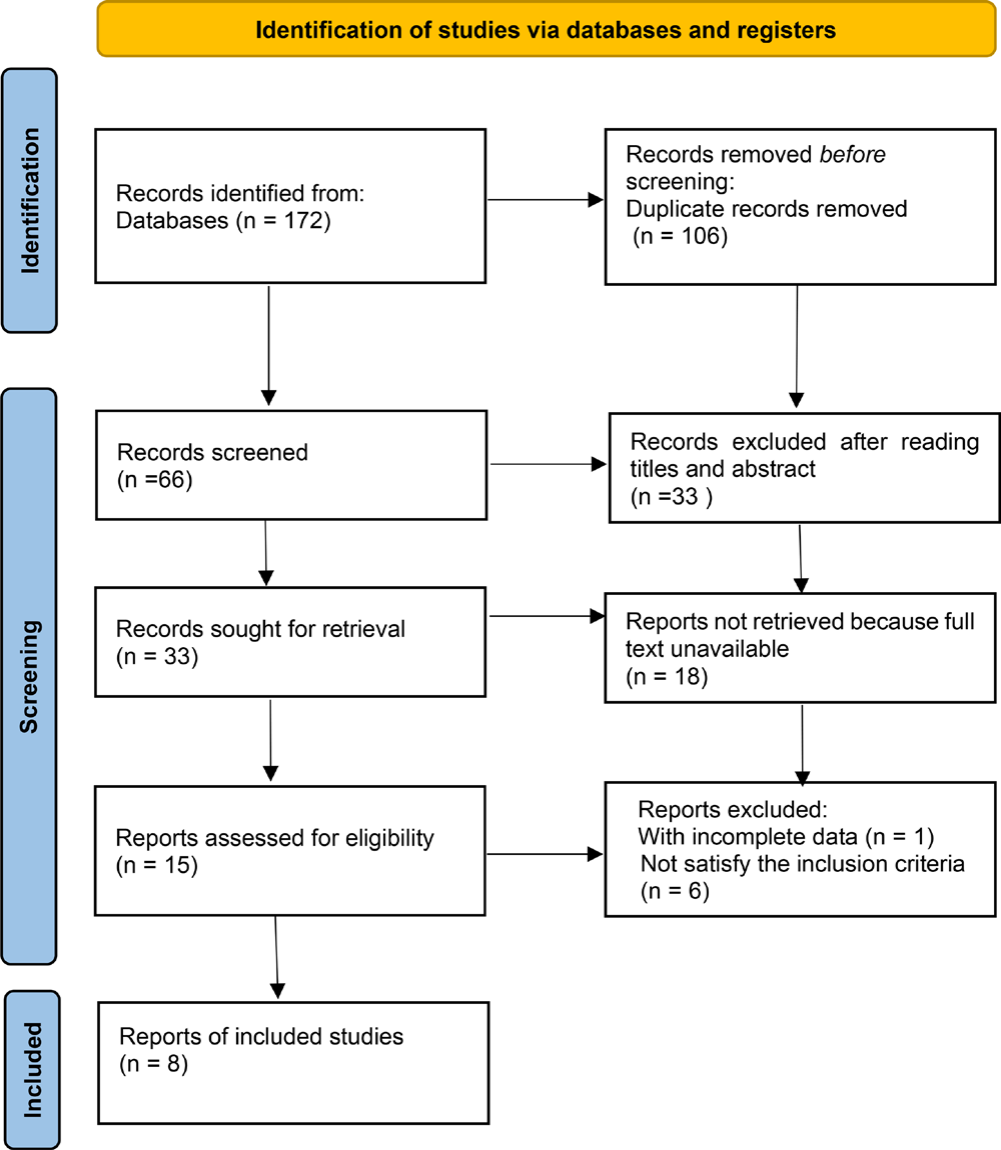
Flow diagram of the systematic review.

As is shown in Figure 1 (PRISMA 2020 flow diagram), after searching 4 databases, 172 trials were found. After eliminating 106 duplicate trials, 33 of the remaining 66 trials were not RCTs, and the remaining 8 trials were RCTs that met the inclusion criteria. Actually, there are 9 trials were finally included for statistical analysis.

In regard to our main metrics shivering, GRADE system grades of evidence are downgraded for the following reasons. First, the risk of bias in 3 studies. Second, initial I^2^ was high (67%) with studies providing a significant risk reduction or a non-significant risk increase. Third, insufficient data to provide comment on precision.. However, because the RR < 0.5, the large effect upgrade the quality of evidence, all studies were designed with randomized methods, quality of efficacy of intravenous acetaminophen on POS was evaluated as low-certainty evidence. Similarly, grades of evidence of severe shivering were moderate (Supplementary Table 2 http://links.lww.com/MD/N184).

Of all the included studies, all of them explored the efficacy of intravenous acetaminophen versus placebo on POS. Among them, 395 cases were treated with acetaminophen and 396 cases with saline. All included documents are from 2014 and later (Table 1). There are 217 cases that received 1000mg of acetaminophen and 461 cases received 15mg/kg of acetaminophen. Summary of postoperative complications including shivering, severe shivering, PON or POV between the acetaminophen and control groups are shown in Table 2.

**Table 1:** Characteristics of the included trials.

Author	Yr	Participants	Type of anesthesia	Type of surgery	Trail	Dosage regimen	Comparisons	Total (case)	Shivering	Severe shivering
Ahmadreza	2016	Adult	SA	Cesarean section	15 min after the delivery of the baby	S	Acetaminophen group 1g IV	55	6	1
							Placebo IV	55	28	27
Amr Samir	2023	Adult	SA/GA	Elective liposuction surgery	30 min before the termination of the procedure	S	Acetaminophen group 1g IV	40	8	8
							Placebo IV	40	26	26
DrU. Sankara	2023	Adult	SA	Lower abdominal surgery		S	Acetaminophen 15 mg/kg IV	30	3	
							Placebo IV	30	21	
Mohammad Sadra	2023	Adult	GA	Inguinal hernia surgery		S	Acetaminophen 0.5 mg/kg IV	40	21	1
							Placebo IV	40	33	5
Takehiro	2019	Adult	SA/GA	Gynecological laparotomy		S	Acetaminophen 15 mg/kg IV	18	4	
							Placebo IV	19	14	
Sanum Kashif	2021	Adult	GA	Septoplasty	20 minutes before completion of surgery	S	Acetaminophen 15 mg/kg IV	30	10	0
							Placebo IV	30	24	5
Gholamreza Khalili	2014	Adult	GA	Upper limbs surgery	Before induction	S	Acetaminophen 15 mg/kg IV	32	5	
							Placebo IV	32	14	
Medha Mohta(A)	2023	Adult	GA	Elective surgery	Immediately after anesthesia induction	S	Acetaminophen 15 mg/kg IV	75	22	
							Placebo IV	75	23	
Medha Mohta(B)	2023	Adult	GA	Elective surgery	30 min before completion of surgery	S	Acetaminophen 15 mg/kg IV	75	9	
							Placebo IV	75	23	

GA = general anesthesia, S = single injection, SA = spinal canal anesthesia.

**Table 2:** Efficacy of acetaminophen on the incidence of perioperative shivering and PON/POV compared with placebo.

Side effects	Number of studies	Acetaminophen	Placebo	RR (95% CI)	I^2^	References
Shivering	9	88/395	206/396	0.43 (0.35,0.52)	67%	[11–18]
Severe shivering	4	15/165	63/165	0.24 (0.15,0.40)	0%	[11,14,16,18]
PON or POV	3	16/190	13/190	1.23 (0.61,2.48)	0%	[11,13,17]

The variables are presented as number of patients, PON = postoperative nausea, POV = postoperative vomiting, RR = pooled risk ratio.

**Figure 2.**
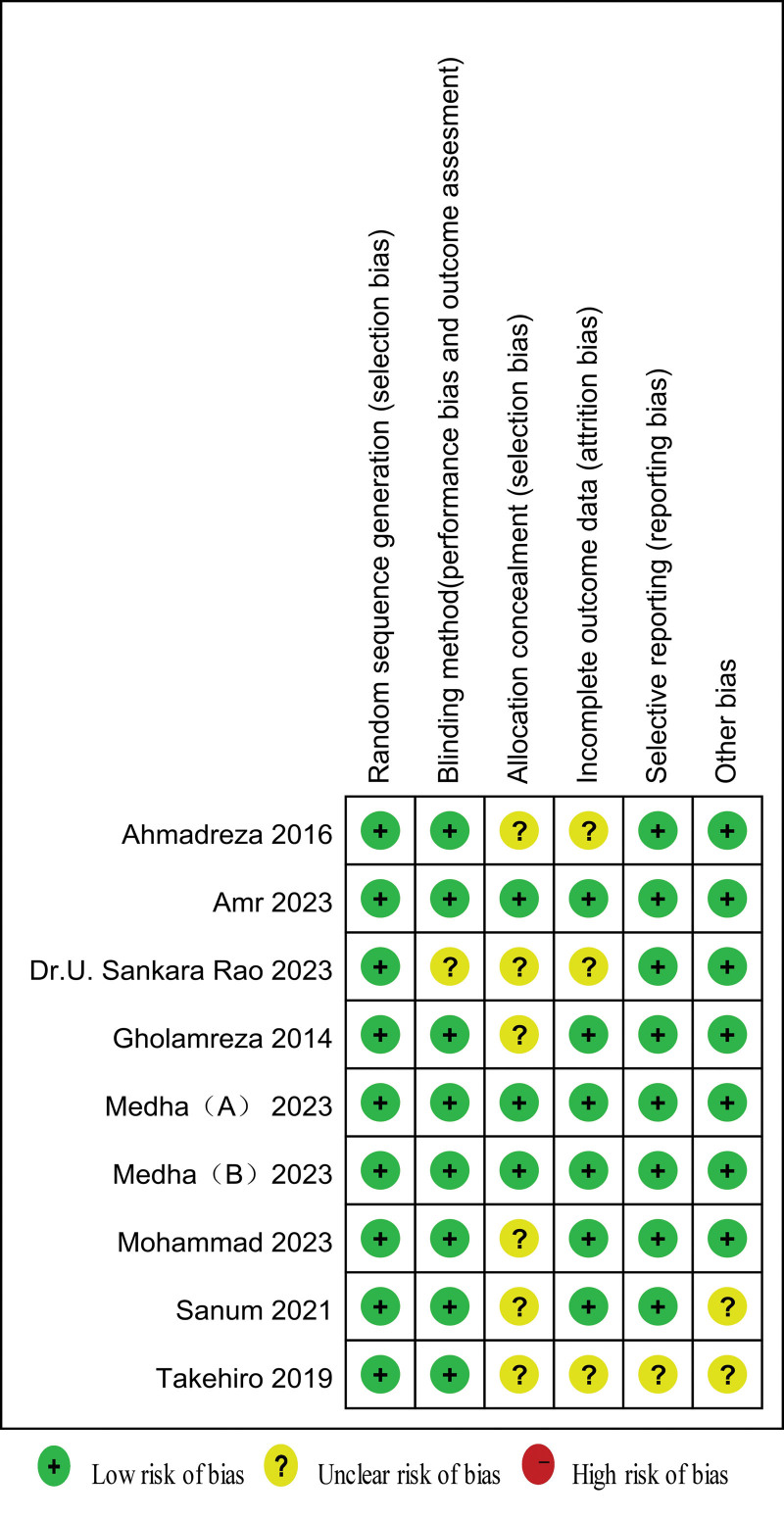
Summary of the risk of bias of the included studies.

As to the assessment of methodological quality, 4 trials reported the endpoints mentioned in the Methods section (reporting bias), without incomplete outcomes (attrition bias) and other biases. A low risk of the overall risk of bias for included 9 trials.^[11–18]^ All 9 included trials provided a detailed description of randomization. The Cochrane Handbook for Systematic Reviews of Interventions was used to evaluate the risk of bias of the RCTs. Eight studies^[11,13–18]^ were double-blinded. Four trials^[11,13,17]^ reported allocation concealment. Most of the studies reviewed lacked sufficient details in allocation concealment, in such cases, we were conservative in our risk of bias evaluation by tending to classify trials as having an “unclear risk of bias.” In addition, all studies reported the completion of the trial without withdrawals, and all the studies reported all the endpoints mentioned in the Methods section (reporting bias). Other biases might exist in 1 trial.^[15]^ An overview of the risk of bias is summarized in Figure 2.

**Figure 3.**
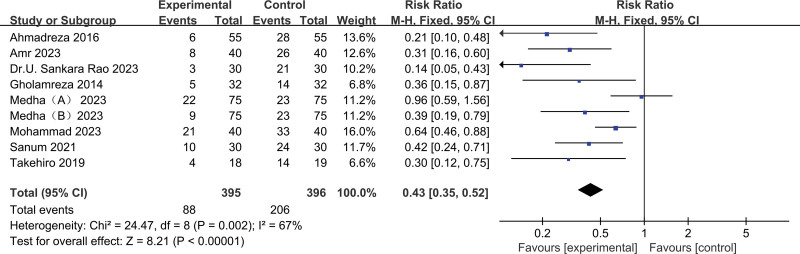
Results of the incidence of postoperative shivering with the application of acetaminophen.

**Figure 4.**
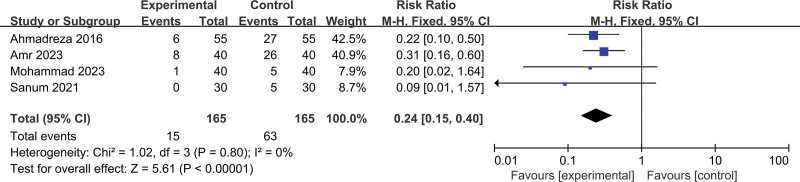
Results of the incidence of severe postoperative shivering with the application of acetaminophen.

**Figure 5.**
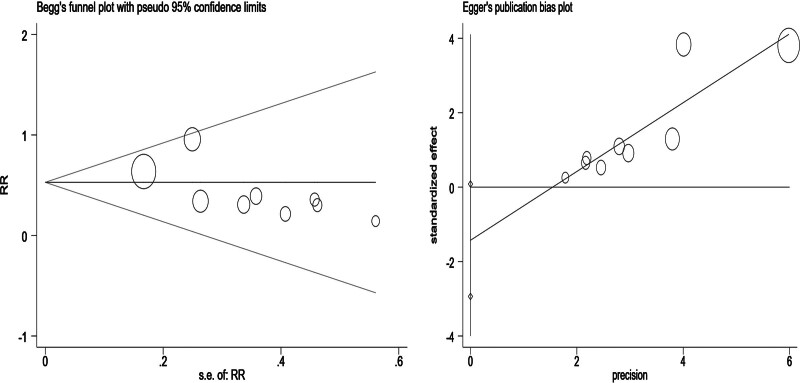
Results of Begg test and Egger test.

Acetaminophen versus placebo on POS: 9 trials,^[11–18]^ the incidence of POS (pooled RR 0.43, 95% CI: 0.35–0.52) in the acetaminophen group was significantly lower than that in the control group (Fig. 3), the reduction effect in severe shivering was also very significant (Fig. 4). Begg and Egger tests suggested no significant publication bias existed in comparisons (Beggers Test *P* = 1.7485, Egger Test *P* = .3991) (Fig. 5). Factors that affected POS were evaluated through further subgroup analysis.

**Figure 6.**
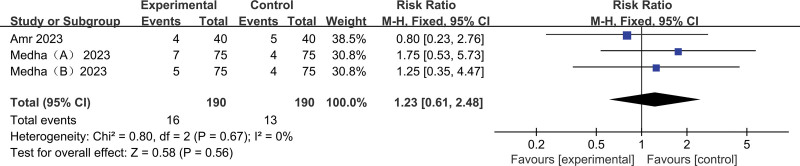
Results of subgroup analysis of the incidence of PON or POV with the application of acetaminophen. PON = postoperative nausea, POS = postoperative shivering.

Other adverse effects: PON (postoperative nausea) or postoperative vomiting (POV): There were 3 studies in 9 trials^[11,13,17]^ reporting PON or POV. Compared with placebo, the experimental group did not show improvement in POS compared to that in the control group, with low heterogeneity (pooled RR 1.23, 95% CI: 0.61–2.48) (Fig. 6).

**Figure 7.**
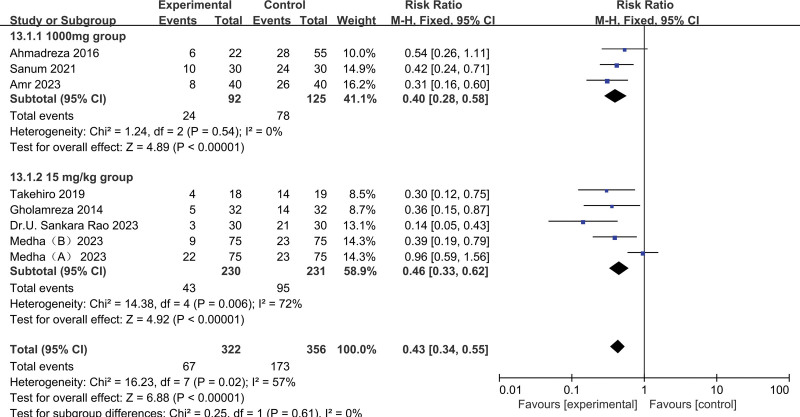
Results of subgroup analysis of the dosage of acetaminophen.

Subgroup analysis: As to the dosage of acetaminophen, not only a single-dose bolus of 1000 mg acetaminophen demonstrated a beneficial effect on POS (pooled RR of 2 trials^[11,16]^: 0.22, 95% CI: 0.10–0.45), but 15 mg/kg acetaminophen also reduced the incidence of POS (pooled RR of 5 trials = ^[12–15]^: 0.43, 95% CI: 0.33–0.62 (Fig. 7).

**Figure 8.**
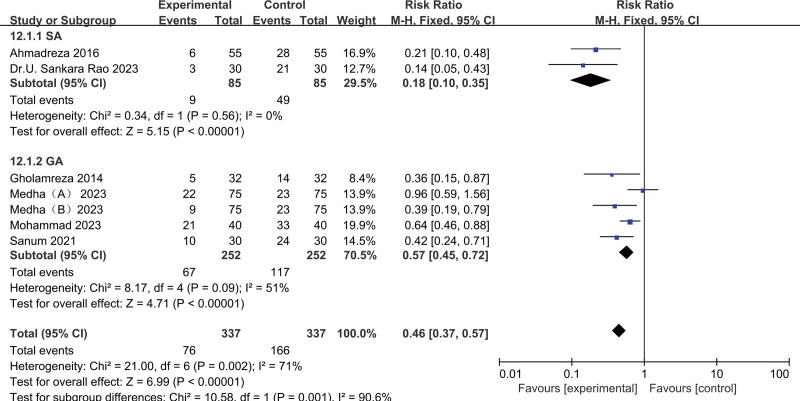
Results of subgroup analysis of types of anesthesia.

Types of anesthesia: Acetaminophen significantly reduced the incidence of POS during both spinal anesthesia (pooled RR of 2 trials^[12,16]^: 0.18, 95% CI: 0.10–0.35) and general anesthesia procedure (pooled RR of 4 trials^[13,15,18]^: 0.57, 95% CI: 0.45–0.72) (Fig. 8).

**Figure 9.**
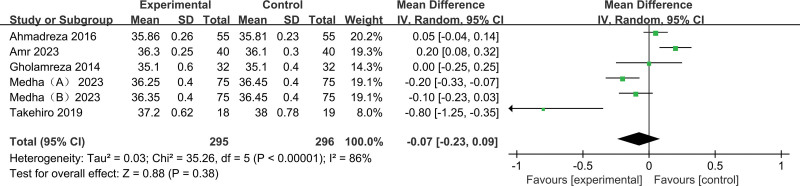
Results of core temperature after anesthesia.

Core temperature after anesthesia: There was no significant statistical difference in core temperature between acetaminophen groups and control groups after anesthesia (pooled RR of 5 trials^[11,13–16]^: −0.07, 95% CI: −1.25 to −0.35) (Fig. 9).

Sensitivity analysis was performed upon the studies with high risk by excluding single study sequentially, but no source of heterogeneity was detected. Meta-regression analysis showed no significant correlations between the year of publication and the type of study design.

## 4. Discussion

Any factors that cause core hypothermia could result in thermoregulatory shivering. Though pharmacologic and nonpharmacologic approaches to reduce perioperative shivering have been explored for decades,^[19]^ solving this tough problem remains a challenge. Thus, finding an effective way proves to be urgent.

The present meta-analysis was undertaken to evaluate the efficacy of prophylactic acetaminophen in the prevention of shivering. The main findings are as follows: Acetaminophen shows clinically and statistically significant superiority to placebo in the prevention of shivering. Acetaminophen was superior to placebo in the prevention of POS without high-risk factors in spinal anesthesia and general anesthesia. Both intravenous 15 mg/kg and 1000 mg bolus infusion have an obvious effect on POS.

The origin of shivering is considered to be posterior hypothalamus and its activity is modulated by inputs from the cold receptors.^[20]^ The COX-2/PGE2 (Prostaglandin E2) pathway is involved in the action of acetaminophen.^[21]^ Research reported that acetaminophen inhibited hippocampal COX-2 expression and PGE2 synthesis through downregulation of the COX-2/PGE2 pathway during the perioperative period.^[22]^ Acetaminophen affects perioperative thermoregulation by inhibiting COX-2, thus allowing the body to avoid a shivering response.^[23]^ The inhibitory effects of acetaminophen on pro-inflammatory mechanisms result in dampened release of pro-inflammatory cytokines during surgery,^[24]^ although its peripheral anti-inflammatory minor effects are relatively limited. Moreover, according to the studies we included, the antishivering effect of acetaminophen may be independent of intraoperative core hypothermia, suggesting that it inhibits thermoregulatory responses by central mechanism.^[25]^

Except for the COX-2/PGE2(Prostaglandin E2) pathway, acetaminophen is metabolized in the brain into AM404(a metabolite that can inhibit the reuptake of anandamide),^[26]^ a known cannabinoid CB1 and CB2 receptor agonist. Postoperative surgical pain makes the non-thermoregulatory shivering happen.^[27]^ The effect of acetaminophen in decreasing postoperative pain makes it also a beneficial method against POS. In addition, acetaminophen has been shown to effectively reduce PON and POV,^[28,29]^ the antiemetic effect was by direct mechanisms or through the reduction in postoperative pain.^[30]^ However, the subgroup studies we included showed opposite results, which may be confirmed by further studies.

Still, this meta-analysis has several limitations. Firstly, the total number of trials included is significant relatively, but the amounts in subgroups, like age, gender, PON or POV, the changes in core temperature before and after anesthesia, are still too little to secure conclusive results. In addition, a lack of risk factors for POS including more intraoperative bleeding, lower core temperature, and higher postoperative pain scores still exists. Therefore, more RCTs, including the kinds of patients and various doses or routes of administration in specific anesthesia or surgeries, should be designed reasonably to detect the efficacy of acetaminophen on POS. According to the GRADE system, the certainty of our findings ranked low across different outcomes, the main limiting factors that contributed to the low quality included the high heterogeneity, the uncertain concealment methods of several studies, and the small sample size of certain researches.

## 5. Conclusion

In conclusion, our present meta-analysis demonstrates that the intravenous prophylactic infusion of acetaminophen has many advantages in lowering perioperative shivering, and the results may provide new evidence to expand the clinical value of acetaminophen in addition to its routine application for the treatment of fever and pain.

## Author contributions

**Investigation:** Xiao Liang, Jikai Liu.

**Methodology:** Qian Cao, Jikai Liu.

**Software:** Jikai Liu.

**Supervision:** Jinfang Zeng, Xiao Liang.

**Writing – original draft:** Jikai Liu, Qian Cao.

**Writing – review & editing:** Jikai Liu, Jinfang Zeng, Xiao Liang.

## Supplementary Material




