# Supplementary material for: Efficacy of intravenous acetaminophen on postoperative shivering: A meta-analysis of randomized controlled trials
Source: Medicine (Baltimore). 2024 Jul 12;103(28):e38710. doi: 10.1097/MD.0000000000038710 (PMC11245272; doi:10.1097/MD.0000000000038710)
Supplement: Supplementary file 1 [file medi-103-e38710-s001.docx]

| **Quality assessment** | | | | | | | **No of patients** | | **Effect** | | **Quality** | **Importance** |
| --- | --- | --- | --- | --- | --- | --- | --- | --- | --- | --- | --- | --- |
|  |  |  |  |  |  |  |  |  |  |  |  |  |
| **No of studies** | **Design** | **Risk of bias** | **Inconsistency** | **Indirectness** | **Imprecision** | **Other considerations** | **Shivering** | **Control** | **Relative (95% CI)** | **Absolute** |  |  |
| **Shivering** | | | | | | | | | | | | |
| 9 | randomised trials | serious^1^ | serious^2^ | no serious indirectness | serious^3^ | none | 88/395  (22.3%) | 206/396  (52%) | RR 0.43 (0.35 to 0.52) | 297 fewer per 1000 (from 250 fewer to 338 fewer) | ⊕⊕OO LOW | CRITICAL |
|  |  |  |  |  |  |  |  | 65% |  | 370 fewer per 1000 (from 312 fewer to 422 fewer) |  |  |

^1^ Three studies had concealment uncertainties. Risk of bias downgraded once.
^2^ Initial I^2^ was high (67%) with studies providing a significant risk reduction or a non significant risk increase. Evidence for this exposure and outcome downgraded once due to Inconsistency.

^3^ Insufficient data to provide comment on precision. Participant number in analyses were <300, unlikely to meet optimal information size parameters. Downgraded once.

| **Quality assessment** | | | | | | | **No of patients** | | **Effect** | | **Quality** | **Importance** |
| --- | --- | --- | --- | --- | --- | --- | --- | --- | --- | --- | --- | --- |
|  |  |  |  |  |  |  |  |  |  |  |  |  |
| **No of studies** | **Design** | **Risk of bias** | **Inconsistency** | **Indirectness** | **Imprecision** | **Other considerations** | **Severe shivering** | **Control** | **Relative (95% CI)** | **Absolute** |  |  |
| **Severe shivering** | | | | | | | | | | | | |
| 4 | randomised trials | serious^1^ | no serious inconsistency | no serious indirectness | serious^2^ | none | 15/165  (9.1%) | 63/165  (38.2%) | RR 0.24 (0.15 to 0.4) | 290 fewer per 1000 (from 229 fewer to 325 fewer) | ⊕⊕⊕O MODERATE | CRITICAL |
|  |  |  |  |  |  |  |  | 32.9% |  | 250 fewer per 1000 (from 197 fewer to 280 fewer) |  |  |

^1^ Three studies had concealment uncertainties. Risk of bias downgraded once.
^2^ Insufficient data to provide comment on precision. Participant number in analyses were <300, unlikely to meet optimal information size parameters. Downgraded once.
