# Supplementary material for: Efficacy of intravenous acetaminophen on postoperative shivering: A meta-analysis of randomized controlled trials
Source: Medicine (Baltimore). 2024 Jul 12;103(28):e38710. doi: 10.1097/MD.0000000000038710 (PMC11245272; doi:10.1097/MD.0000000000038710)
Supplement: Supplementary file 2 [file medi-103-e38710-s002.docx]

|  | RCT subgroup | |
| --- | --- | --- |
| **Primary outcomes** | **Pooled results [95% Cl]** | **GRADE recommendations** |
| Postoperative shivering | (0.35 to 0.52) | ⊕⊕OO **LOW** |
| Severe Postoperative shivering | (0.15 to 0.4) | ⊕⊕⊕O **MODERATE** |
| PON/POV | (0.61 to 2.48) | ⊕⊕OO **LOW** |
| Hypotension | (0.35 to 4.51) | ⊕⊕OO **LOW** |
| Anesthesia types | (0.19 to 0.39) | ⊕⊕⊕⊕ **HIGH** |
| Dose of acetaminophen | (0.2 to 0.39) | ⊕⊕OO **LOW** |

Cl=confidence interval, RCT = randomized controled trial， PON= postoperative nausea，

POV= postoperative nausea and vomiting

**Supplementary Table1-Summary of findings table and GRADE recommendations.**
